# Investigation of the Effect of Alkyl Chain Length on the Size and Distribution of Thiol-Stabilized Silver Nanoparticles for Proton Exchange Membrane Fuel Cell Applications

**DOI:** 10.3390/membranes16020058

**Published:** 2026-02-02

**Authors:** Md Farabi Rahman, Haoyan Fang, Aniket Raut, Aaron Sloutski, Miriam Rafailovich

**Affiliations:** Department of Materials Science and Chemical Engineering, Stony Brook University, Stony Brook, NY 11794, USA; haoyan.fang@stonybrook.edu (H.F.); aniket.raut@stonybrook.edu (A.R.); aaron.sloutski@stonybrook.edu (A.S.)

**Keywords:** Ag NPs/Nafion interface, PEMFCs, CO resistance, catalyst, alkyl chain length, catalytic activity, distribution of nanoparticles

## Abstract

This article reports on how the length of the alkyl chain influences the morphological properties of thiol-stabilized silver nanoparticles (Ag NPs) and their subsequent effects on the performance and durability of proton exchange membrane fuel cells (PEMFCs). We synthesized thiol-stabilized Ag NPs by varying the alkyl chain length: 1-hexane thiol (C6), 1-octanethiol (C8), 1-decanethiol (C10), 1-dodecanethiol (C12), and 1-tetradecanethiol (C14), which we achieved using the two–phase Brust–Schiffrin method. X-ray Diffraction (XRD) patterns confirm the formation of crystalline Ag NPs. A morphological study conducted using a Transmission Electron Microscope (TEM) demonstrated that smaller alkyl chain length thiols (C6, C8, and C10) tend to coalesce, while C12 shows better uniformity with no agglomeration. C14 produces larger nanoparticles. A distinct pressure-area isotherm was observed when Ag NPs were spread at the water/air interface of a Langmuir–Blodgett (LB) trough. After obtaining the monolayer formation pressure range, we coated the Nafion 117 membrane of a polymer electrolyte membrane fuel cell with these nanoparticles to form monolayers of different Ag NPs (C6, C8, C12, C14) at various surface pressures (2 mN/m, 6 mN/m and 10 mN/m). Maximum power output enhancement was observed for C12, while other nanoparticles (C6, C8, C10, C14) did not exhibit noticeable power enhancement for PEMFCs. C12 Ag NPs deposited at surface pressure 6 mN/m give maximum power density increase (26.5%) at the fuel cell test station. In addition, we examined the carbon monoxide (CO) resistance test by mixing 0.1% CO with hydrogen (H2), and C12 Ag NPs showed the highest resistance to CO poisoning. However, no enhancement in power or CO tolerance was observed when C12 Ag NPs were coated by spray coating. These outcomes showcase that alkyl chain length plays a critical role in controlling the size and distribution of thiol-stabilized nanoparticles, which eventually has a direct impact on the performance and CO resistance of PEMFCs when applied to polymer electrolyte (Nafion 117). In addition, surface pressure during monolayer formation controls the distribution of Ag NPs (the distance between nanoparticles at the membrane interface), which is necessary to achieve catalytic activity for power improvement and to prevent platinum (Pt) poisoning by CO oxidation at ambient conditions.

## 1. Introduction

**Figure 1:**
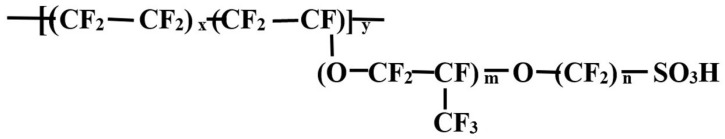
Chemical structure of Nafion.

PEMFCs are one of the most promising areas in sustainable energy. They have various useful properties, including high energy efficiency, a comparatively low working temperature, their status as a green alternative to fossil fuel-based electricity generation, their flexibility and scalability for various applications, their relative compactness and light weight, easy and fast fueling and start-up options, quiet operation, etc. [1,2,3,4,5,6]. Platinum (Pt) is used as a catalyst in PEMFCs, but it is expensive and susceptible to CO poisoning [7,8,9]. CO forms CO-Pt bonds at catalytic sites that inhibit Pt-H_2_ catalysis. H_2_ is the fuel of this technology, primarily produced by steam reforming from hydrocarbons, which have 1–2% CO [10,11,12,13]. A Pt catalyst can be poisoned by the presence of even a minimal amount of CO, which reduces the performance of the PEM fuel cell substantially (the presence of 25 ppm of CO decreases the current density by 50%), and ultra-purification of H_2_ is complicated and costly [14,15]. Furthermore, the generation of pure H_2_ gas by electrolysis is not viable due to costs and electricity use, given its environmental impact [16,17]. Green synthesis of H_2_ via photocatalysis is currently being studied and has yet to be commercialized [18,19]. Although operating temperatures above 100 °C can minimize Pt poisoning caused by CO oxidation, PEMFCs’ performance and durability decrease significantly due to Nafion dehydration, which remains the commercially employed electrolyte in this technology [20]. In addition, several factors contribute to the degradation of PEMFCs during long-term use, including electrode material degradation, catalyst loss, and mechanical damage to the membrane electrode assembly (MEA) [21]. Nafion is the commercial brand name of an ion exchange membrane, which chemically consists of perfluorosulfonic acid (PFSA) copolymer (Figure 1). A tetrafluoroethylene backbone with added sulfonic acid groups exhibits unique properties, including thermal and chemical stability, high proton conduction rates, and longer lifetimes, making it a promising membrane for applications such as hydrogen fuel cells and electrolyzers [22,23]. However, the Nafion electrolyte in PEMFCs loses its lifetime if dehydration occurs due to shape changes caused by crack formation, as the Nafion membrane is confined between the gas diffusion layers. It develops stress patterns in the MEA through plane cracks [24,25].

Researchers have investigated alternative solutions to overcome these limitations, including oxygen (O_2_) bleeding, voltage/current pulsing, and elevated working temperature; however, these strategies have long-term stability issues, are difficult to install, and may pose safety concerns [26,27,28,29]. Consequently, catalyst optimization is necessary to mitigate this CO poisoning drawback by oxidizing CO. Previously, CO oxidation was demonstrated at lower temperatures by leveraging synergistic effects between metal catalysts and metal oxide supports. Haruta et al. first demonstrated that titania (TiO_2_)-supported gold nanoparticles (Au NPs) can oxidize CO at low temperatures [30]. An explanation for this effect is provided by Neurock and Yates, who suggest that Au NPs have an oblate morphology with a stepped interface at the contact line with TiO_2_ [31,32]. According to their study, Au NPs are atomically supported by neighboring TiO_2_, which activates O_2_ molecules through chemical bonding to both Au and Ti^4+^ catalytic sites. The apparent activation energy is found to be only 0.16 eV for CO oxidation at 120 K and around 0.2–0.3 eV at higher temperatures of 200–300 K. Subsequently, different metal oxide supports, such as CeO_2_, Al_2_O_3_, or FeOx, were employed to test this [33,34,35]. Although this technology shows promising results for CO oxidation at lower temperatures, it has limitations with regard to its application in PEMFCs because metal oxides require either a high deposition temperature (above 100 °C) or an electrochemical process, both of which can harm the Nafion membrane and increase the risk of film nonuniformity.

In our previous report, we described a novel, easy method for depositing Au NPs with controllable particle spacing onto a Nafion electrolyte at ambient temperature [36]. The LB method was used to deposit a uniform layer of Au NPs at the air–water interface at room temperature. Au NPs were prepared using the Brust–Schiffrin method, where a two-phase synthesis technique is used to develop thiol-stabilized metal NPs [37]. The fuel cell’s performance was enhanced by more than 50% under ambient conditions. We observed that enhancement occurs with a monolayer of Au NPs without a metal oxide (TiO_2_) layer. In addition, substantial CO resistance was found in these Au particles at room temperature. In this study, we investigated the suitability of silver (Ag) NPs for PEMFCs, focusing on performance and durability. Ag as a catalyst exhibits great promise due to its properties, such as thermal stability, a lack of toxicity, low cost, easy availability, and a high activity level [38,39,40,41]. Moreover, some properties of Ag at the nanoscale differ from its bulk form, including exceptionally high catalytic activity at low temperatures. Therefore, we selected a relatively cheap Ag metal catalyst and synthesized Ag NPs according to the Brust–Schiffrin method [42]. We used five different alkylthiols—C6, C8, C10, C12, and C14—as stabilizing agent for nanoparticles, and their chemical structure and formula are listed in Table 1. We observed that alkyl chain length plays a significant role in preventing nanoparticles from agglomerating. Furthermore, we observed that Ag NPs, free of agglomeration and deposited by the LB method at the monolayer level, possess active catalytic sites for CO oxidation, demonstrating resistance to Pt poisoning during PEMFC operation at ambient temperature and an increase in performance (current density and power density). Moreover, in this report, we sought to unravel the mechanism underlying this phenomenon: the role of alkyl chain length on silver nanoparticles’ morphological characteristics and the subsequent consequences when interface engineering is performed between these particles and the Nafion electrolyte in a PEM fuel cell. Finally, we proposed a catalytic mechanism for the enhancement effect observed with C12 Ag NPs and the significance of nanoscale interface design. According to our research, this is the first report of the Ag NPs/Nafion interface design via LB deposition for PEMFC applications.

## 2. Experimental Design

### 2.1. Chemicals and Materials

All materials were of analytical grade and were used without further refinement. Silver nitrate (AgNO_3_), tetraoctylammonium bromide (TOABr), C6, C8, C10, C12, and C14, sodium borohydride (NaBH_4_), and toluene were bought from Sigma-Aldrich, Burlington, MA, USA. We procured ethanol from PHARMCO-AAPER, Houston, TX, USA; Nafion 117 from Ion Power, Tyrone, PA, USA; Pt/C (40 wt.%) and the electrode with 0.1 mg cm^−2^ Pt loading from FUEL CELL Store, Bryan, TX, USA. Nafion ionomer solution (15 wt.%) was obtained from Fuel Cell Earth LLC, Woburn, MA, USA. Linde Inc., Danbury, CT, USA supplied H_2_ (99.999%), N_2_ (99.9%), O_2_ (99.8%), and 3.1% CO in H_2_ (99.98%) gases.

### 2.2. Fabrication of Ag NPs

**Figure 2:**
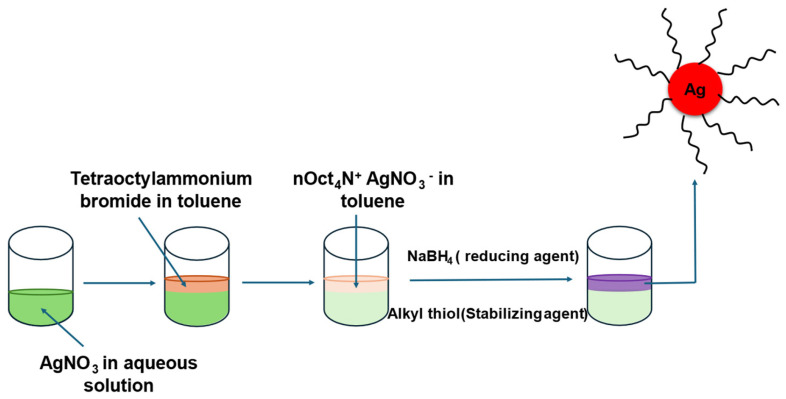
Synthesis of silver nanoparticles (Ag NPs) by the Brust–Schiffrin method.

The two-phase Brust–Schiffrin method (Figure 2) was adopted to prepare thiol-stabilized Ag NPs, in which an aqueous solution of AgNO_3_ (1.0 mmol in 36 mL of H_2_O) was mixed with a solution of TOABr in toluene (4.8 mmol in 96 mL of toluene). The two-phase mixture was stirred vigorously for 20 min, and 200 microliters of each thiol (C6, C8, C10, C12, and C14, respectively) was then added. Aqueous solution of NaBH_4_ (11.97 mmol in 30 mL of water) was added slowly under stirring, and the solution turned brown immediately. The mixture was stirred for 3 h, and then the organic phase was concentrated to 5 mL in a rotary evaporator. All the reaction steps mentioned above were executed at ambient conditions (room temperature). Afterwards, 200 mL of ethanol was added to the 5 mL concentrated solution to eliminate excess thiol, and the mixture was kept in a refrigerator at 4 °C overnight. Finally, the precipitate was centrifuged, washed with ethanol three times, and dried in a vacuum desiccator for 2 days. Ag NPs prepared from C6, C8, C10, C12 and C14 are designated as C6, C8, C10, C12 and C14 Ag NPs respectively.

### 2.3. Deposition of Ag NPs

**Figure 3:**
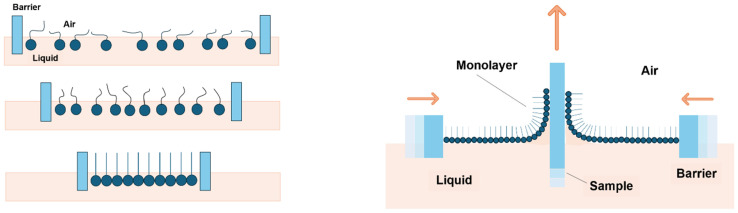
Langmuir–Blodgett principle and deposition technique.

The LB trough (Model KSV NIMA 300, Biolin Scientific, Phoenix, AZ, USA) method was used to deposit Ag NPs. Toluene was used as a solvent to prepare a Ag NPs solution (1 mg/mL), which was sonicated for at least 1 h before use. Nafion 117 membrane was immersed in distilled water (DI) within the LB trough prior to the deposition of Ag NPs. Afterwards, using a glass syringe, 250 μL of the Ag NP solution was dispensed onto the water, and toluene was allowed to evaporate for 15 min. First, from the isothermal curve, the monolayer formation pressure range was determined, and Ag NPs were eventually deposited at different surface pressures within this range. Figure 3 depicts the LB trough principle and coating technique, where nanoparticles are deposited at the air–water interface on the Nafion membrane.

In addition to the LB technique, we employed the direct spray method with a Ag NP concentration of 0.1 mg cm^−2^ using an air spray gun.

### 2.4. Crystal Structure and Morphology Study of Ag NPs

Structural property of Ag NPs was examined by X-ray Diffraction (XRD) (Model MiniFlex, Rigaku, Tokyo, Japan) under Cu Kα radiation (1.54 Å) from 10° to 80° at a scan rate of 5° min^−1^.

Morphological properties of these Ag NPs were characterized by Transmission Electron Microscope (TEM) (Model JEM 1400, JEOL, Peabody, MA, USA). To determine particle size and inter-particle distance, the ImageJ bundled with 64-bit Java 8 software was used.

### 2.5. PEMFC Demonstration Kit Performance

Initially, we conducted a fuel cell performance test at room temperature with a kit having a maximum power output of 200 mW. The membrane electrode assembly (MEA) of the demonstration kit system was constructed by Nafion 117 membrane sandwiched between two platinum (Pt) electrodes (Pt loading of 0.1 mg cm^−2^) with a surface area of 16 cm^2^. The Nafion membrane was hydrated for 5 min by submersion in DI. Uniform pressure was applied across the MEA using a four-point assembly, and the hydrogen flow rate was maintained at 60 SCCM using a previously optimized flow meter.

### 2.6. High-Power PEMFC Performance Test

A fuel cell performance test was also conducted at the Fuel Cell Test Station obtained from Fuel Cell Technologies, Inc., Albuquerque, NM, USA. We prepared catalyst ink by stirring a mixture of Pt/C, Nafion ionomer, and 2-propanol. Afterwards, the ink was sprayed onto microporous carbon paper measuring 5 cm^2^ using a spray gun with N_2_ gas. 0.01 mg/cm^2^ Pt loading was applied to both the anode and cathode by drying the gas diffusion electrodes on a hot plate at 85 °C. A performance test was conducted with 99.99% pure H_2_ flowing at 60 SCCM at the anode and 100 SCCM of O_2_ at the cathode. Both gases were humidified to 100% relative humidity (RH) and heated to 5 °C above the operating temperature to prevent any condensation at both the cathode and anode. At first, we conducted control tests at three temperatures (30 °C, 60 °C, and 90 °C). After optimizing the temperature, we ran all the samples at the optimized temperature. Table 2 presents an outline of the test conditions at the fuel cell test station.

### 2.7. CO Tolerance Testing for Ag-Modified Catalysts

To evaluate the effect of Ag nanoparticles (Ag NPs) on CO tolerance, a CO resistance test was performed using a PEMFC demonstration kit. A gas mixture containing 3.1% CO in pure H_2_ was diluted to 0.1% CO in H_2_, which was then used as the anode feed gas. The test was conducted following the same procedures and operating parameters as those used during the PEMFC kit performance testing.

### 2.8. Durability Study

An accelerated stress test (AST) was conducted using a potentiostat (Biologic P200, Knoxville, TN, USA) to evaluate MEA durability by cycling the potential between 0.6 and 0.9 V vs. RHE at a scan rate of 50 mV s^−1^. The AST was performed under an H_2_/N_2_ atmosphere at 80 °C, 2 h. stabilization, 100% relative humidity, and atmospheric pressure.

## 3. Results and Discussion

### 3.1. XRD Analysis

**Figure 4:**
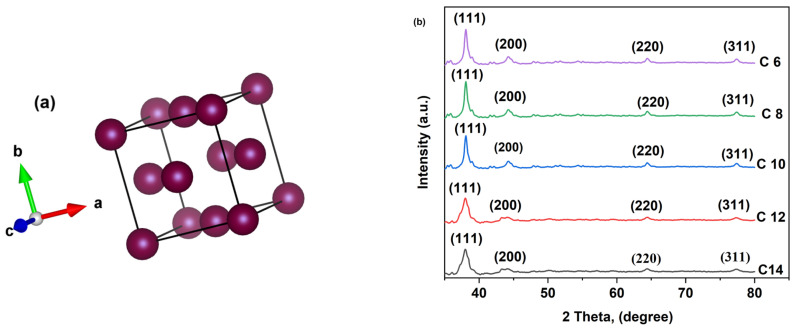
(**a**) Crystal structure of face-centered cubic (fcc) Ag NPs, (**b**) XRD patterns of the synthesized Ag NPs.

Figure 4 shows the XRD patterns of the prepared nanoparticles; all (C6, C8, C10, C12, C14) indicate polycrystalline Ag NPs with a face-centered cubic (Fcc) phase. The associated diffraction peaks are (111), (200), (220), and (311), and Ag NPs showed a strong preferential orientation towards (111), indicating the polycrystallinity of the samples. However, for C12 and C14 Ag NPs, peak broadening of (111) occurs because of prominent nanocrystallinity compared to other Ag NPs (C6, C8, C10).

Furthermore, the crystallographic parameters (crystal system, space group, lattice parameters, unit cell volume) of these nanoparticles are summarized in Table 3.

We determined the crystallite size (Scherrer equation) and lattice strain from the (111) peak of the XRD pattern of Ag NPs (Table 4). C12 and C14 Ag NPs have low crystallite sizes owing to their nanocrystallinity, and high lattice strain arises from their low atomic coordination numbers [30].

### 3.2. Morphology Study of Ag NPs by TEM

**Figure 5:**
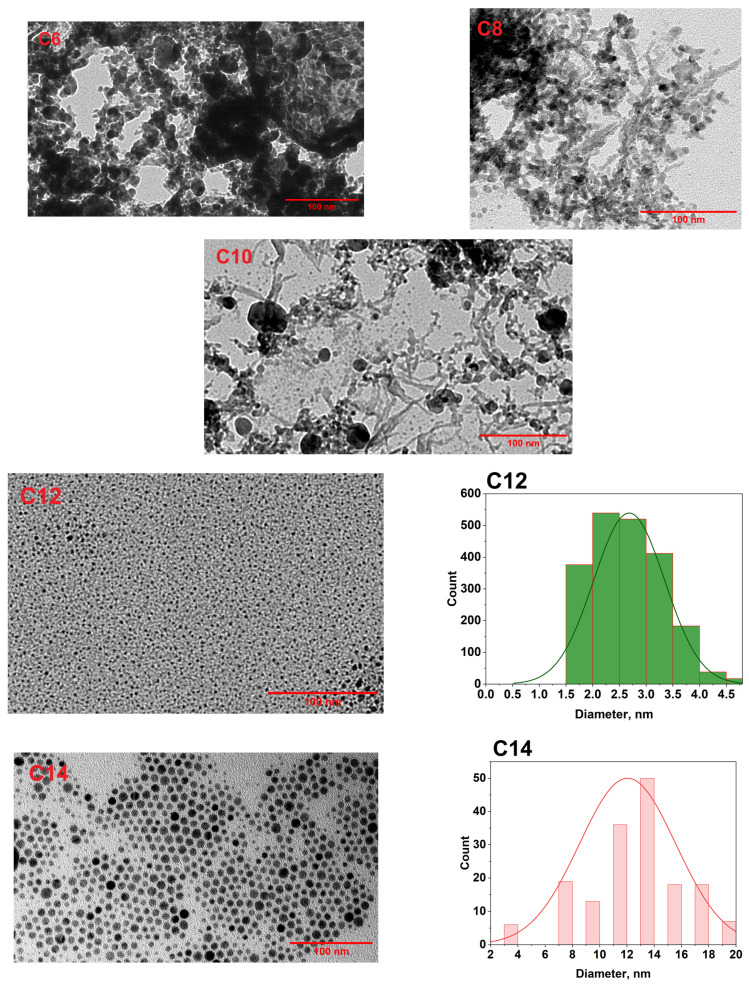
TEM images of Ag NPs synthesized using different alkyl thiols and the size distribution of C12 and C14 Ag NPs.

Ag NPs prepared employing five different thiols were examined by TEM to perceive their morphological properties. From Figure 5, it is evident that alkyl chain length has a controlling effect on the morphology of nanoparticles during their synthesis. Low-chain-length alkyl thiols (C6, C8, C10) tend to aggregate; indeed, shorter carbon chains produce a less effective protective layer and closer packing, making metal nanoparticles more vulnerable to coalescence [43,44,45,46]. C12- and C14-Ag NPs exhibit well-dispersed nanoparticles. We measured the size distributions of these two types of nanoparticles, with average particle sizes of 2.68 ± 0.66 nm and 12.04 ± 0.87 nm for the C12 and C14 samples, respectively. A longer alkyl chain on thiol molecules produces a higher steric repulsive force among the nanoparticles, resulting in more dispersed nanoparticles. Furthermore, longer alkyl chains produce larger nanoparticles, and their stability stems from a robust, insulating coating provided by a longer carbon chain [47,48,49].

### 3.3. Ag NP Film Development

**Figure 6:**
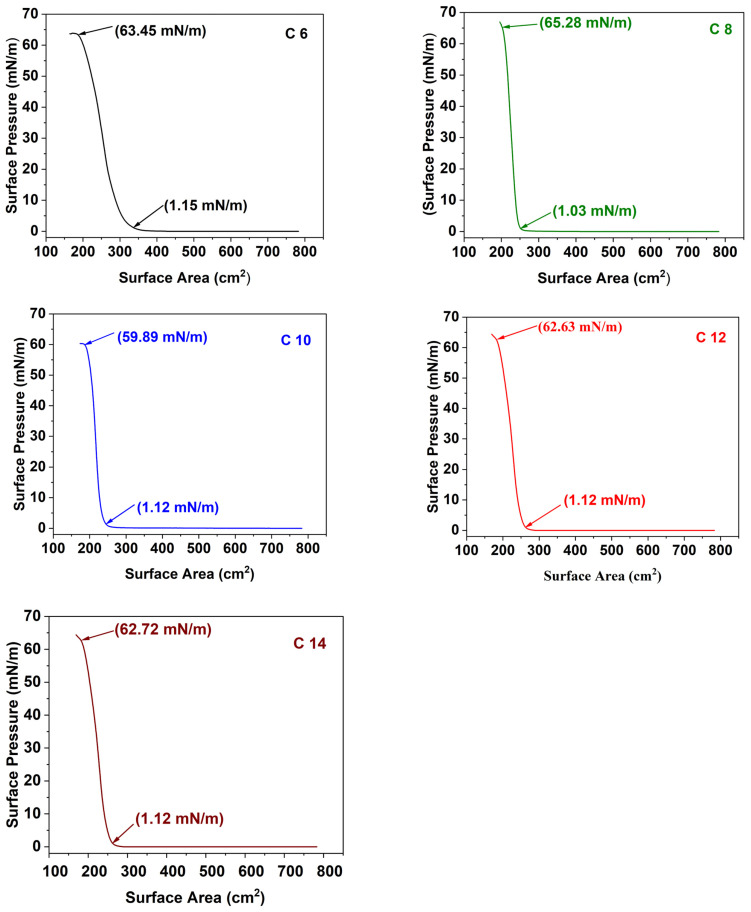
Isothermal curve for alkyl thiol-stabilized (C6, C8, C10, C12, and C14) Ag NPs.

Monolayers of Ag NPs were deposited on the Nafion membrane following the LB trough method. At first, we derived a pressure–area isotherm for all the Ag NPs (Figure 6) when they were spread on the air–water interface in an LB trough system. Figure 6 shows that monolayers form between 1.03 and 1.15 mN/m surface pressure for all five Ag NPs, with a maximum around 60 mN/m. By exploiting this technique, Ag NPs were deposited onto a Nafion membrane at three different surface pressures (2 mN/m, 6 mN/m, and 10 mN/m). By varying surface tension, the number of nanoparticles at the Nafion membrane interface can be tuned to the monolayer level.

### 3.4. PEMFC Performance Test

**Figure 7:**
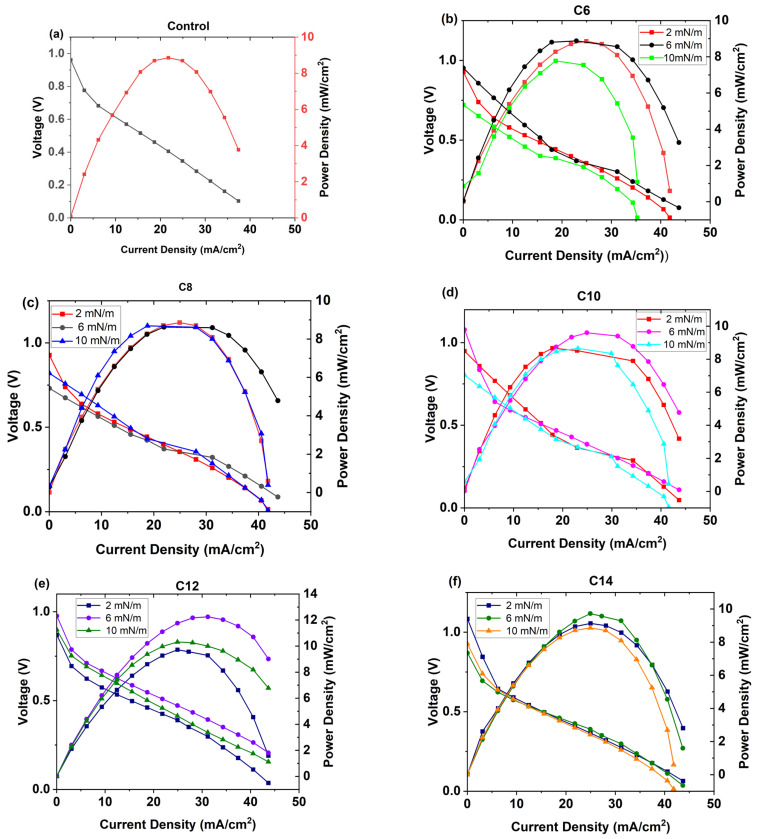
Polarization and power curves for (**a**) control, (**b**) C6 samples, (**c**) C8 samples, (**d**) C10 samples, (**e**) C12 samples, and (**f**) C14 samples were determined based on the fuel cell kit demonstration.

**Figure 8:**
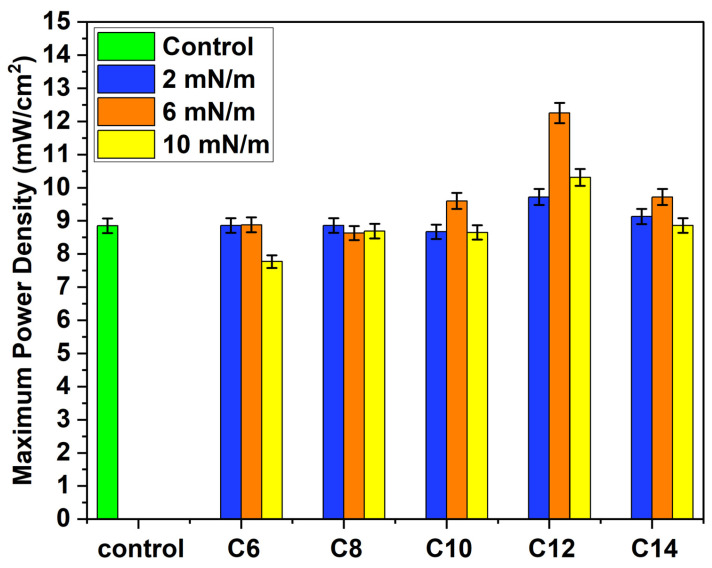
Maximum power output obtained by the different samples at the demonstration kit fuel cell.

At first, we determined polarization and power curves using a demonstration kit for all Ag NPs (C6, C8, C10, C12, and C14) deposited on a Nafion membrane by the LB method at three different surface pressures (2 mN/m, 6 mN/m, and 10 mN/m). Figure 7 shows that C12 Ag NPs exhibit the most significant power output enhancement. Among C12 samples, Ag NPs deposited at 6 mN/m demonstrate a maximum power density increase of 38.42%, while samples deposited at surface pressures of 2 mN/m and 10 mN/m exhibit maximum power output increases of 9.83% and 16.5%, respectively. Furthermore, C14 samples display a similar enhancement trend: C14 2, 6, and 10 mN/m samples demonstrate maximum power density increases of 3.16%, 14.6%, and 8.86%, respectively. On the contrary, C6, C8, and C10 Ag NPs do not show any noticeable improvement after depositing at similar conditions (surface pressures) on Nafion electrolyte. The maximum power density of all the samples is shown in Figure 8.

**Figure 9:**
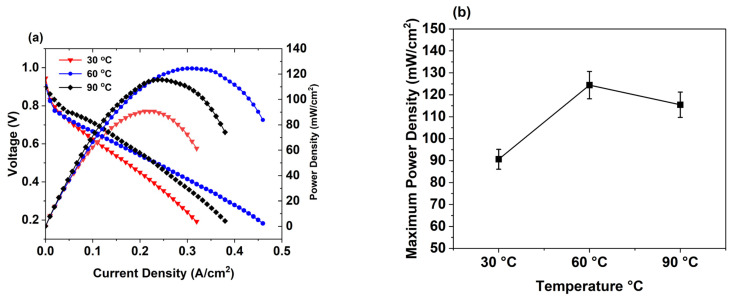
(**a**) Polarization and power curves for control and (**b**) maximum power density values at 30 °C, 60 °C, and 90 °C.

We further conducted MEA test at a professional fuel cell test station to evaluate the results obtained in the demonstration kit cell, which can also be translated there. Initially, we optimized the operating temperature for this fuel cell test station. We performed polarization and power tests with control at three different working temperatures: 30 °C, 60 °C, and 90 °C (Figure 9). At a lower temperature (30 °C), the lowest maximum power output value of the control is observed. This is because at lower temperatures, the Pt catalyst suffers from reduced activity, severe ohmic polarization, and increased proton impedance, which reduces conductivity and durability in the PEMFC [50,51]. At 60 °C, the highest value of maximum power density is obtained, while at 90 °C, the performance of control slightly decreases compared to that at 60 °C owing to dehydration of the Nafion membrane. Nafion begins to lose bulk and loosely bound water above 80 °C, leading to a decrease in proton conductivity and performance [52,53].

**Figure 10:**
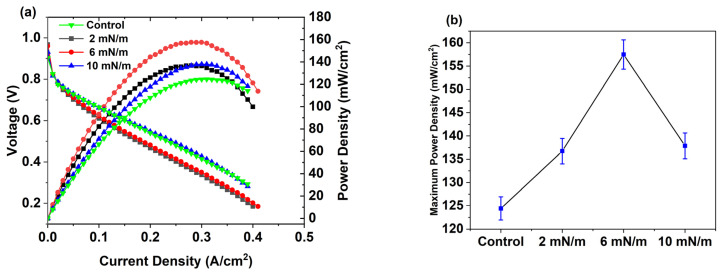
(**a**) Performance curves (current density and power), (**b**) comparison of maximum power density of samples prepared from C12 Ag NPs at different surface pressures (2, 6, 10 mN/m) and control at the fuel cell test station.

After optimizing the working temperature to 60 °C, we conducted performance (current density and power) tests at the fuel cell test station for C12, with samples prepared at three different surface pressures: 2, 6, and 10 mN/m. The performance graphs are shown in Figure 10. Several substantial differences are noted among the samples. All three C12 Ag NPs samples show enhancement of power: among them, the 6 mN/m sample exhibits the best performance (maximum power enhancement of 26.5%), whereas the 2 mN/m and 10 mN/m samples have 9.89% and 10.8% of power rise, respectively. The same trend is also observed for current density.

The above outcome elucidates the significance of Ag NP morphology in controlling PEMFC performance. From TEM images, we observe that alkyl chain length controls the shape and growth of nanoparticles; agglomerated nanoparticles (C6, C18, C10) do not affect PEMFC performance when applied to a Nafion membrane using an LB trough at different surface pressures within the monolayer formation range. On the other hand, C12 Ag NPs are free of agglomeration and showed the most significant increase in power. Furthermore, C14 Ag NPs are larger and exhibit higher current density and power, but at lower levels than those of the C12 samples. Moreover, professional fuel test station data corroborates the findings from our experiments with C12 samples. The outcomes also reveal that Ag NPs have a catalytic effect on performance, boosting it in polymer electrolyte fuel cells when they are free of clustering and have a larger surface area (smaller particles). Moreover, this enhancement is crucial given the polarization losses that develop during PEMFCS operation [54].

**Figure 11:**
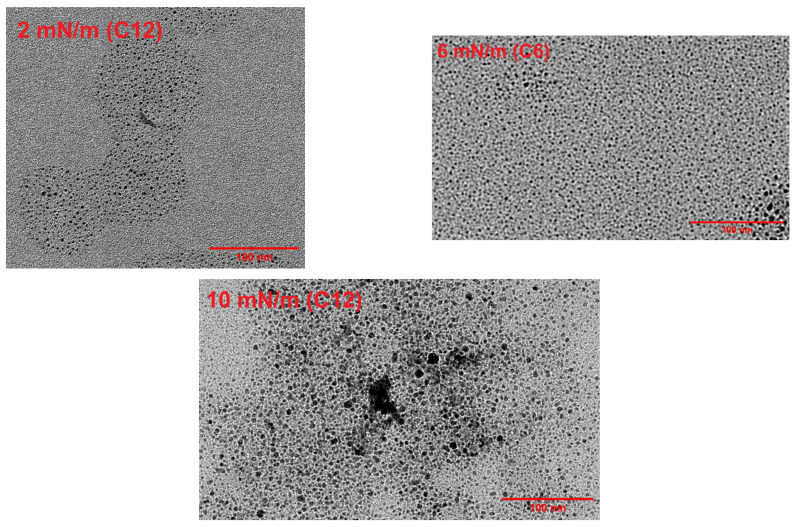
TEM images of C12 Ag NPs at surface pressures 2, 6, 10 mN/m, respectively, on the LB trough.

Another important finding to be noted that surface pressure is pivotal in designing the interface between polymer electrolyte (Nafion) and Ag NPs using the LB technique: we observe that among the three (2, 6, 10 mN/m) surface pressures MEA developed from 6 mN/m coated membrane results in the maximum performance advancement. To understand this behavior further, we collected nanoparticles at surface pressures of 2 mN/m, 6 mN/m and 10 mN/m for C12 to appraise the 2D pattern of nanoparticles by lifting TEM grids at those pressures. Figure 11 shows that at low pressure (2 mN/m), the monolayer coverage was incomplete, with vacancies. At a surface pressure of 6 mN/m, the nanoparticles were distributed in a closely packed, homogeneous order. However, the film collapsed at a pressure of 10 mN/m. The interparticle distance was 2.41 ± 0.57 nm at a surface pressure of 6 mN/m. Such a design of C6 Ag NPs at a surface pressure of 6 mN/m at the Nafion interface yielded the highest catalytic activity of Ag NPs due to the high catalytic surface area.

**Figure 12:**
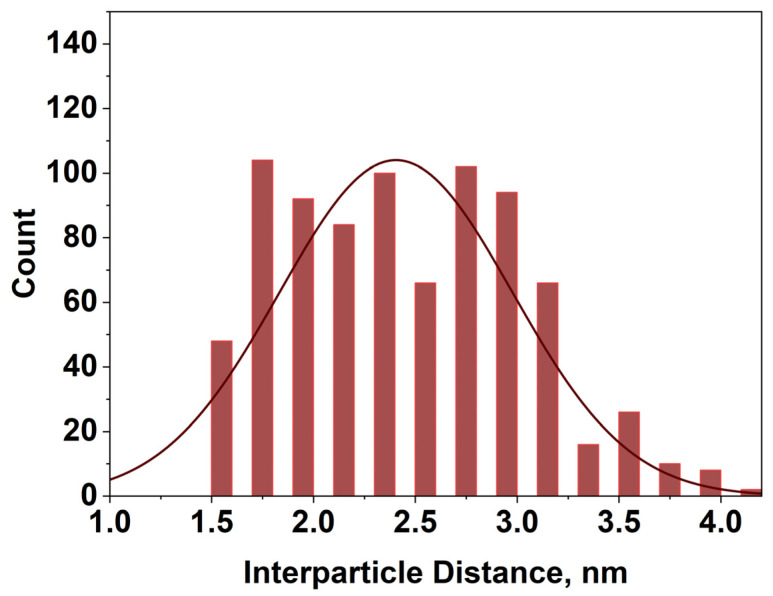
Inter-particle distance of C12 Ag NPs at surface pressure 6 mN/m on LB trough.

We also determined the edge-to-edge interparticle distance for C12 Ag NPs at a surface pressure of 6 mN/m using ImageJ software. Figure 12 shows that the average interparticle distance was 2.41 ± 0.57 nm.

### 3.5. CO Resistance Test

**Figure 13:**
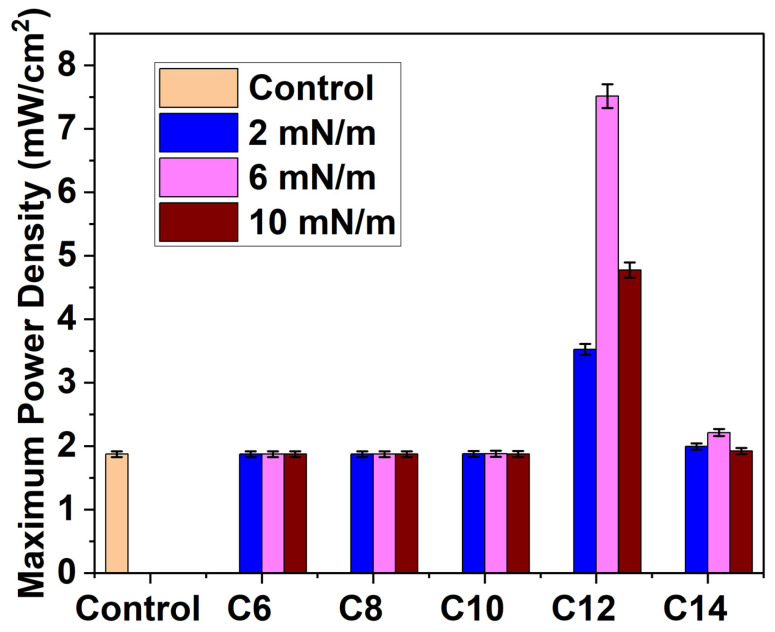
Maximum power density bar graph for all samples evaluated at ambient conditions with 0.1% CO in H_2_.

We performed a CO tolerance test in which 0.1% CO-containing H_2_ was fed to the anode. Figure 13 illustrates the maximum power output value under this condition and the corresponding comparison among the samples. The most significant effect of interface engineering between Ag NPs and Nafion membrane is realized in this experiment. Ag C12 NPs coated at 6 mN/m surface pressure show maximum resistance against CO poisoning of a Pt catalyst and the maximum power output escalates to 300% compared to the control, followed by an 88% and 154% improvement in C12 samples deposited at 2 and 10 mN/m surface pressure, respectively. C14 samples show an upward trend, similar to C12, but the values are not significant. On the contrary, lower alkyl chain length thiol-stabilized Ag NPs do not show any resistance against CO. We note that Ag NPs can promote oxidation of CO at ambient temperature when the particles have a larger surface area (which is free from aggregation and smaller in size). In addition, the arrangement of nanoparticles on the membrane surface controls the catalytic activity of Ag NPs. Also, 2 mN/m C12 Ag NPs do not have full coverage on the Nafion interface, and at a higher pressure, with a 10 mN/m distance among the particles, the distance is reduced, and little aggregation occurs. On the other hand, owing to a relatively homogeneous distribution of Ag NPs at surface pressure 6 mN/m, an ideal arrangement of Ag NPs with an interparticle distance of 2.41 ± 0.57 nm is revealed, which leads to CO oxidation and increases the lifetime of the Pt catalyst. Smaller particles have more corner atoms or steps for catalysis, and the interdistance among particles is crucial for allowing CO to become trapped for oxidation. At low nanoparticle coverage (2 mN/m), catalytic activity is lower due to a small number of C12 Ag NPs. At 6 mN/m surface pressure, we achieve complete coverage of C12 Ag NPs with a homogeneous distribution of nanoparticles and an interparticle distance of 2.41 ± 0.57 nm, allowing CO to be trapped and oxidized at low temperature in the presence of C12 Ag NPs. At higher surface pressure (10 mN/m), even though the number of Ag NPs is high, monolayers start to collapse. Moreover, owing to the presence of a high number of Ag NPs, there is not enough room for CO to settle at the surface and oxidize [55].

### 3.6. Durability Study

**Figure 14:**
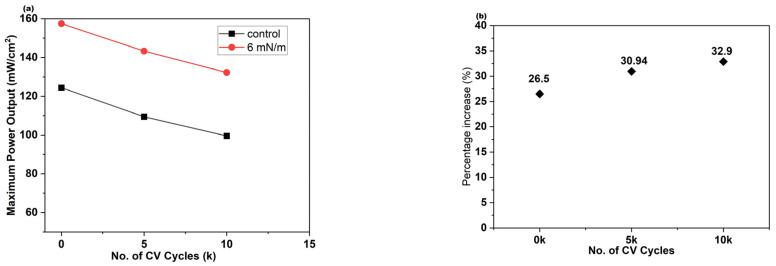
(**a**) Maximum power output for control and C12 Ag NP deposited at 6 mN/m after 0 k, 5 k, and 10 k cycles. (**b**) Maximum power density increase (%) of C12 Ag NPs at surface pressure of 6 mN/m with respect to control (uncoated MEA) after 0 k, 5 k, and 10 k cycles in AST.

We evaluated the durability of the control and C12 Ag NPs deposited at 6 mN/m using an accelerated stress test (AST) cycling protocol approved by the DOE. As the number of cycles increased, the maximum current density decreased for both MEAs. The decrease in maximum power with cycle number is shown in Figure 14, where the coated MEA exhibits a sustained performance enhancement of approximately 26–33% throughout the test. These outcomes reveal that the performance enhancement associated with the coated membrane is maintained for at least 10 k AST cycles.

### 3.7. Performance and CO Resistance Test of C12 Ag NPs Coated by the Spray Method

**Figure 15:**
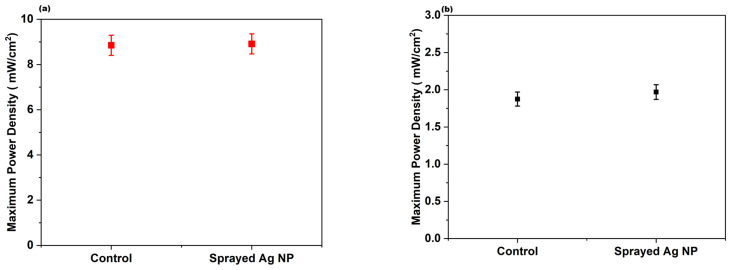
(**a**) Maximum power output for control and C12 Ag NPs deposited by the spray method. (**b**) Maximum power density when fed with 0.1% CO at the anode for control and C12 Ag NPs coated by the spray method.

C12 Ag NPs were applied on Nafion membrane by the spray method, and the concentration was 0.1 mg cm^−2^. Figure 15 shows that no power enhancement occurred and that no CO resistance was observed. Indeed, the significance of LB coating on Nafion at a certain pressure is reflected by this result. In addition to morphology, the arrangement of nanoparticles at the nanoparticle–electrolyte (Nafion) interface is vital to PEMFC performance and CO tolerance.

### 3.8. Catalytic Mechanism

**Figure 16:**
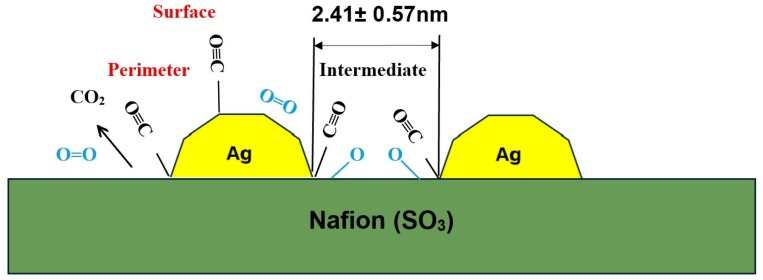
Proposed model for the catalytic mechanism of CO oxidation at the Nafion/Ag NP interface.

It has been well established that Au nanoparticles (NPs) supported on metal oxides are highly effective catalysts for CO oxidation reactions [30,31]. CO is unavoidably generated during the routine operation of PEMFC, and its oxidation is the rate-limiting step due to CO poisoning of the Pt catalyst. In our previous study, we demonstrated that enhanced power output and improved CO tolerance were observed only when the membrane was coated with oblate Au nanoparticles [36]. This behavior was attributed to the role of the Nafion polymer, which was found to function analogously to adsorbed reactants or metal oxide supports. Smaller nanoparticles (1–5 nm) have low coordination numbers, especially on their edges and corners. The interaction between small Ag nanoparticles and Nafion is modeled by considering the adsorption of an SO_3_-containing group located at the termini of Nafion side-chains (Figure 16). We consider synthesized C12 Ag NPs to possess an oblate shape with steps, and CO molecules are adsorbed on the edges or corners of the Ag NP surface. The assumption is based on previous research, which concluded that for low-temperature CO oxidation, nanoparticles should be smaller (below 5 nm) and have more corner atoms (oblate shape) [30,36]. Our results indicate that the SO_3_ species provided by Nafion play a crucial role in facilitating CO oxidation. Specifically, SO_3_ species are strongly adsorbed at low-coordinated Ag sites via their three terminal oxygen atoms, enabling effective oxidation of CO to CO_2_ at the nanoparticle perimeter. Consequently, the overall CO concentration is significantly reduced.

The activation barrier for oxidizing adsorbed CO in the presence of SO_3_ (SO_3_ + CO → SO_2_ + CO_2_) is substantially lower than that for CO removal via the water–gas shift reaction (WGSR, H_2_O + CO → CO_2_ + H_2_), which is energetically unfavorable in the absence of SO_3_. In our model, SO_3_ adsorbed on Ag nanoparticles serves as a simplified representation of the Ag–Nafion interaction. Notably, the interaction between SO_3_ and Ag is relatively weak, allowing facile removal of an oxygen atom from SO_3_ to oxidize CO. This interaction is expected to be further weakened when the full Nafion side-chain ligands are considered (Ag–SO_3_–R). In contrast, if SO_3_ binds too strongly to Ag, CO oxidation by SO_3_ becomes energetically hindered due to the high stability of the Ag–SO_3_ complex. Therefore, direct interaction between Ag nanoparticles and Nafion membranes is essential for facilitating CO oxidation and alleviating CO poisoning. In this synergistic system, Ag nanoparticles act as the active oxidation catalyst, while Nafion serves as an efficient oxidant source. The interparticle distance plays a crucial role in CO oxidation: higher interparticle distances lead to fewer available sites for CO oxidation, and particles which are too close together are not suitable for reaching Ag–SO_3_–R interfaces.

## 4. Conclusions

In summary, we have demonstrated that, for thiol-stabilized nanoparticles, alkyl chain length acts as a surfactant, controlling nanoparticle morphology. For shorter chain lengths (C6, C8, and C10), our synthesized Ag NPs exhibited instability and agglomeration. All the nanoparticles possessed a typical Ag Fcc crystal structure; however, C12 and C14 Ag NPs exhibited broadening of the (111) XRD peak, reflecting their well-dispersed nanoscale nature. All the fabricated particles spread at the air–water interface in the LB trough, showing a pressure-area isotherm. No significant performance enhancement was observed for any of the C6, C8, C10, and C14 Ag NPs samples when they were deposited in the LB trough in the monolayer formation range. The C12 samples showed substantial performance improvement when coated at 6 mN/m surface pressure, while 2 mN/m and 10 mN/m pressure resulted in growth at a lower scale. We also conducted performance tests on C12 samples at a professional fuel cell test station, where we observed the same performance trend. To achieve the catalytic effect of Ag NPs in PEMFC applications, the particles’ morphology significantly influenced their catalytic activity. The particles should be free of clustering, and their size should not be too large to yield a low surface area. We also investigated CO resistance with Ag NPs and found that C12 samples coated at a surface pressure of 6 mN/m exhibited the highest resistance (a 300% increase in power compared to the control). In addition to the morphology of the Ag NPs, we realized the significance of nanoparticle distribution at the monolayer level on the Nafion electrolyte. At surface pressure 6 mN/m, the Ag NPs were homogeneously distributed on Nafion, and a certain distance among inter-particles (2.41 ± 0.57 nm) was necessary to promote CO oxidation on the Ag NP interface. During the durability study, 6 mN/m C12 Ag NPs showed a similar enhancement compared to the uncoated sample. In addition, C12 Ag NPs, when spray-coated, did not show improved performance or CO resistance. We proposed a model for the catalytic mechanism of CO oxidation, and an HR-TEM study, along with DFT calculations, will reinforce it.

## Figures and Tables

**Table 1:** Molecular formula and chemical structure of the alkyl-thiols employed in this research.

Name	Molecular Formula	Chemical Structure
1-Hexanethiol (C6)	C_6_H_14_S	CH_3_–(CH_2_)_5_–SH
1-Octanethiol (C8)	C_8_H_18_S	CH_3_–(CH_2_)_7_–SH
1-Decanethiol (C10)	C_10_H_22_S	CH_3_–(CH_2_)_9_–SH
1-Dodecanethiol (C12)	C_12_H_26_S	CH_3_–(CH_2_)_11_–SH
1-Tetradecanethiol (C14)	C_14_H_30_S	CH_3_–(CH_2_)_13_–SH

**Table 2:** MEA test parameters at fuel cell test station.

Pt Load (mg/cm^2^)	Cell Area (cm^2^)	Temperature (°C)	Relative Humidity (RH%)	Flow Rate		Back Pressure	
				Anode (SCCM)	Cathode (SCCM)	Anode (kPa)	Cathode (kPa)
0.1	16	30, 60, 90	100	60	100	150	150

**Table 3:** Crystallographic parameters of the fabricated Ag NPs.

Crystal System	Space Group	Lattice Parameters		Unit Cell Volume, V (Å)^3^
		a = b = c (Å)	α = β = γ	
Face-centered Cubic	F m-3 m	4.08550	90°	68.1923

**Table 4:** Crystallite size and lattice strain (%) of the prepared Ag NPs.

Type of Ag NP	Crystallite Size (nm)	Lattice Strain (%)
C6	13.75	0.008
C8	13.6	0.008
C10	12.71	0.009
C12	2.83	0.039
C14	2.95	0.039

## Data Availability

The data that support the findings of this study are available from the corresponding author upon reasonable request.
